# A call to enhance transparency among Egyptian medical schools

**DOI:** 10.1186/s12909-023-04464-1

**Published:** 2023-06-30

**Authors:** Wagdy Talaat, Mariam Asaad Amin, Mohamed Reda Bassiouny, Nancy Husseiny Hassan, Omayma Hamed

**Affiliations:** 1 Medical Education Department, Suez Canal Faculty of Medicine, Egyptian Society for Medical Education (ESME), Ismailia, Egypt; 2 Anatomy Department, Ain Shams Faculty of Medicine, Egyptian Society for Medical Education (ESME), Cairo, Egypt; 3 Pediatrics Department; Mansoura Faculty of Medicine, Egyptian Society for Medical Education (ESME), Mansoura, Egypt; 4 Anatomy Department; Zagazig Faculty of Medicine, Egyptian Society for Medical Education (ESME), Zagazig, Egypt; 5 Medical Education, Armed Forces College of Medicine, Egyptian Society for Medical Education (ESME), Cairo, Egypt

**Keywords:** Transparency, Quality, Accreditation, Accountability, Website, NAQAAE

## Abstract

**Background:**

Making accreditation results easily accessible has become *a worldwide* essential issue, especially after international standards were created for medical education. The Egyptian Society for Medical Education (ESME) expects Egyptian medical schools to be more open about their accreditation results to build trust with students, families, and the community. This will help ensure newly graduated doctors are of high quality. Our literature review found almost no information on how transparent Egyptian medical school websites are with posting their accreditation results. Students and families use these websites to choose schools and be confident in the quality of education, so accreditation results should be easily accessible.

**Methods:**

This study was conducted to estimate the information transparency of Egyptian medical colleges’ websites regarding their accreditation process. Twenty-five official websites of Egyptian medical colleges, as well as official website of the National Authority for Quality Assurance and Accreditation of Education (NAQAAE) were reviewed. The websites’ search considers two main criteria for transparency. Each criterion is further divided into several information items. Data was recorded and analyzed using Research Electronic Data Capture software (REDCap). The authors excluded, from the data analysis, newly established schools of less than five years of age that were not required to apply for accreditation yet.

**Results:**

The results of the research showed that only thirteen colleges registered their credentials on their websites. However, the amount of data available about the process, dates, and documents was very limited. Accreditation information for these thirteen schools is confirmed by information on the NAQAAE website. Other information on other important elements such as accountability and future plans was almost completely missing. Conclusion: The authors concluded that due to the lack of basic information on the websites of Egyptian medical schools about their institutional accreditation status, serious steps should be taken by medical schools and the National Accreditation Authority to encourage openness and ensure transparency towards institutional accreditation.

## Background

Quality assurance and accreditation has always been a challenge for higher education institutions to ensure their appropriate standing among their peers, both locally and internationally. A medical educational institution or medical training program is evaluated by an accredited body using a set of published standards and pre-established protocols as part of the external quality assessment process for the field of medicine [1]. In 2020, the World Federation for Medical Education (WFME) made it clear that decisions to accredit medical programs should be made publicly available. Furthermore, the publication of self-study and accreditation reports that provide the basis for decisions should not remain a secret for others to learn from [2]. In addition, an examination of the institutional curriculum as well as its learning and teaching methods is mandatory to ensure that students are taught effectively and thus ensure that they are responsible for optimal patient care at a later date [3].

In Egypt, medical schools undergo a five-year accreditation cycle by the National Authority for Quality Assurance and Accreditation of Education (NAQAAE) [4]. A mandatory part of the accreditation requirements is making the corporate vision, mission, and strategic goals publicly available. It is well recognized that the quality of education is best delivered within the context of the institution’s mission as well as its educational programs [5]. In the era of national reform of the entire medical curricula; Accreditation reports are important to establish the actual status of each college [6].

The present study investigates the existing level of transparency in disclosing information by Egyptian medical institutions and puts forward recommendations to enhance their openness and clarity in sharing information.

The concept of transparency is defined in different ways depending on the context. Transparency has also been proposed as an alternative to regulations, especially in political spheres. Some argue that making information readily available is a more cost effective and effective strategy compared to imposing strict rules and controls. They believe transparency can replace excessive government intervention [7]. The phrase “abuse of entrusted power for private gain” is a frequent definition of corruption used by Transparency International (TI), a non-governmental organization (NGO) that works to combat it globally. But corruption in higher education also includes “lack of academic integrity” [8]. Scholars agree on the fact that transparency is beneficial to an organization; It allows the public to obtain information about the establishment and management of a particular entity [9].

The internationalization of medical education has increased the number and diversity of providers of higher education programs both nationally and globally. This has made it more difficult for students to choose where and what to study to a level that may require it to be customized according to their needs, plans and capabilities. It is the concept of transparency in providing information across platforms to students as well as to the public that would help overcome such problems.The Egyptian Association for Medical Education (ESME) aspire that Egyptian medical faculties work more towards transparency and openness to obtain more effective feedback [10]. It is a striking note that most medical schools in Egypt, both before and after accreditation, share minimal information about curriculum mapping, accreditation process, or outcomes on their websites. Our current research spans the two key transparency criteria of Egyptian medical schools. Thus, the purpose of the current study was to investigate the official websites of 25 medical schools in Egypt as well as the website of the National Authority for Quality Assurance and Accreditation of Education (NAQAAE).

## Methods

From October to December 2021, we independently reviewed twenty-five official websites of Egyptian medical schools as well as the NAQAAE website to search for two main criteria for transparency (having a website addressing full accreditation information and comprehensiveness of credentials). Each criterion was divided into several information items (as listed in Table 1).

**Table 1:** Disclosure of the most important criteria and sub-criteria used to measure transparency in medical colleges in Egypt

Criteria
Having a website with full credential information
⇒ Website optimized for search engines
⇒ At least a section on the Institute’s website is entirely dedicated to displaying accreditation information
⇒ Results from accreditation processes are plain language descriptions
⇒ Regular updates (within 6 months)
⇒ Availability of English translation for foreign students
⇒ Availability of a research tool and/or discussion board that relates to the credentials.
The comprehensiveness of the credentials/information on the website which includes:
⇒ Current accreditation status and accreditation history
⇒ Syllabus outline
⇒ Vision
⇒ Mission
⇒ Goals/objectives
⇒ Values
⇒ Competencies
⇒ Self-study
⇒ Annual reports
⇒ KPIs: institutional, programs & courses
⇒ Student’s Independent Opinion
⇒ Social accountability
⇒ Resources (Human, Material, Technical and Financial)
⇒ Future plans/reform
⇒ Full contact information

The WFME standards guide institutions to appropriately assess their educational practices within their local contexts [11]. We selected some elements that are directly related to the topic of transparency and openness in medical education. The authors modified the components of the accreditation framework by excluding some aspects of information about accreditation criteria that have weak relevance to our study. Aspects of each information item are recorded as present (such as website text or downloadable document) or nonexistent. Data were recorded in the Electronic Data Search Center (REDCap) [12] hosted on the Egyptian Neonatal Network server [13]. To analyze each information item across all medical schools, we tabulated descriptive statistics for all accreditation information items across the twenty-five schools.

Throughout this study, the authors decided to add additional information items that could correlate and explain the transparent position adopted by different medical schools. These criteria are as follows:

- Determine whether the school has clearly demonstrated the capabilities that their students should acquire at the conclusion of their program and how the school is implementing evaluation of these reached competencies.
- Schools’ self-studies on home page in a clear presentation.
- Annual reports and their updates till the date of the study.
- Social Accountability, or the ability of medical schools to address community health needs and priority health problems in all education, research, and service domains. It indicates these institutions’ capacity to design instruction in a way that has the greatest positive influence on the health of the community [14]. These details should be made obvious to students on medical schools’ websites.
- Unbiased student opinion through student surveys regarding the curriculum, facilities, and institutional educational activities.
- Resources: human, physical, technical, and financial resources.
- Surveys that were conducted to determine whether medical schools are prepared with future plans or reforms to improve their efficacy and graduate educational level.

## Results

Out of the twenty-five schools surveyed, twenty-three belong to public universities and two are private. Twenty-three of these medical schools optimized their websites for search engines, while the other two medical schools required more than one search engine to find their website. All websites show the history profile of the medical school. By viewing the year the schools were founded, the authors were able to identify medical schools that had been established for less than 5 years. These newly established schools were excluded from the data analysis since they had not yet applied for accreditation.

**Fig. 1:**
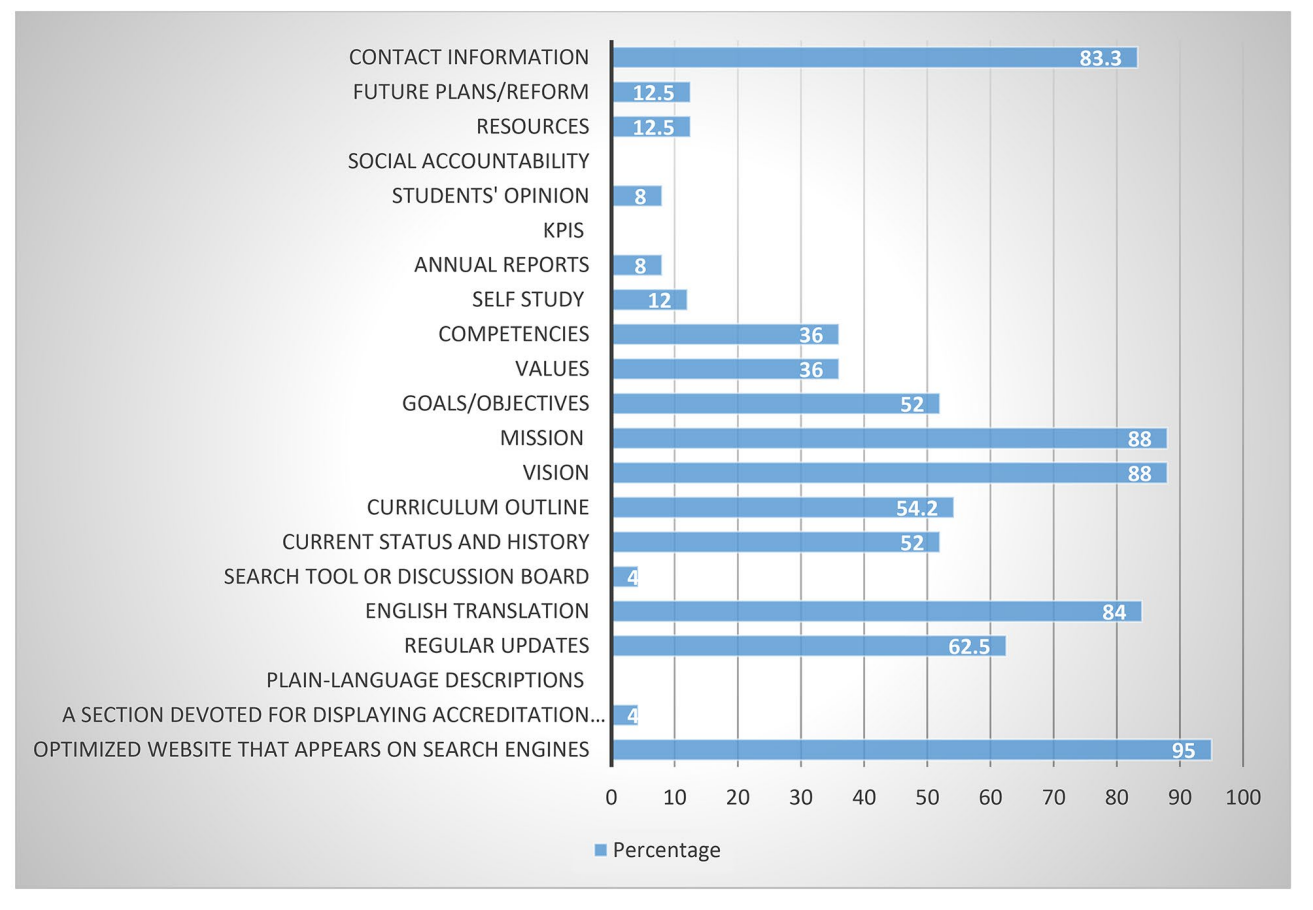
Shows the availability of data on medical school websites

Websites are translated into English for international students in only twenty-one schools. The websites of the remaining schools only appear in Arabic, and there is even an inactive website that displays the error every time the authors try to access it. Also, fifteen schools have updated their websites regularly and only twenty schools offer complete contact information about their staff and management for any inquiries (Fig. 1).

With respect to vision and mission statements and publicly displaying this information, 22 medical colleges listed their vision and mission statements publicly. Specifically, 13 medical schools published their goals while only 9 disclosed the values adopted by their leadership and administration (Fig. 1). Clearly defined and well-structured vision and mission statements articulate an organization’s purpose and foster a sense of belonging among administrators and faculty. When present, these statements define the path to success that everyone in the organization must follow. The vision and mission must be communicated to stakeholders to project the organization’s image. Additionally, they guide the development of strategic goals and values that are properly designed [15].

One of the most surprising findings was that 54.2% of Egyptian medical schools did not disclose their curriculum outlines or teaching and learning methods to students. Only 45.8% provided curriculum outlines, but these were part of broader university regulations rather than in a separate, easily accessible section of their websites. However, some still adhere to the old bylaws for Egyptian medical colleges without auditing their websites since 2016. With technological advancements, websites have proven to be a virtual window for potential applicants lacking prior information about undergraduate or postgraduate programs. However, there are discrepancies between what applicants need and what medical school websites actually provide [16].

Authors decided to include additional information items that relate to and interpret the transparent stance taken by various medical schools. Accordingly, the findings of this study in this regard were as follows:

a- Only nine websites mentioned the 2017 National Academic Reference Standards set by the NAQAAE for the development of the new curriculum in Egyptian medical schools. However, none of the websites provide details on how these competencies will be implemented or measured using key performance indicators at the institutional, program, or course levels. The websites only give general information about the competencies without outlining specific implementation plans or expected key performance indicators.

b- Three schools reveal their self-studies in section B. Two of which are absent from the school’s home page and buried under the quality assurance unit achievements.

c- Annual reports: Only two out of the twenty-five medical colleges made their annual reports publicly available. Furthermore, there were no new updates as the reports were dated from 2018 to 2019.

d- With respect to social accountability or the ability of medical schools to address societal health needs, our review of Egyptian medical schools’ websites found that none of the colleges indicated any social responsibility activities on their websites. Only one university was recognized by an external database for being socially responsible, while the other schools’ websites contained no information regarding social accountability initiatives or community engagement efforts [15].

e- Unbiased student opinion: Only the results of student surveys regarding the curriculum, facilities, and institutional educational activities were available on the websites of two medical schools.

f- Resources: Three medical schools gave brief summaries of their human, physical, technical, and financial resources. Only a complete directory of the academic faculty and their accomplishments was available for one of these schools.

g- On several school websites, a survey was conducted to determine whether medical schools are prepared with future plans or reforms to improve their efficacy and graduate educational level. It is remarkable that only three institutions presented any ideas for institutional infrastructure and patient service transformation. However, no mention was made of any educational reform data that would have an impact on accreditation.

## Discussion

The Ministry of Higher Education’s website emphasizes Egypt’s sustainable development plans until 2030 where the strategic goals fully aim to improve educational systems to align with international standards. This will increase competitiveness in educational institutions that can guarantee the best educational outcomes [17]. To achieve these objectives, the medical curriculum in Egypt was reformed in 2017 for all medical schools. However, these revised curricula do not appear to be openly displayed on the schools’ websites in a transparent manner that would facilitate feedback from the public or stimulate healthy competition among medical schools as envisioned by the Ministry’s goals. Rather, the curricula seem to be communicated to students on an annual basis to serve immediate educational needs. Greater transparency in publicly presenting the updated curricula could help align the schools’ practices more closely with the Ministry’s strategic goals. Since transparency is a declaration of the accountability of medical schools towards the society they serve, enhancing the transparency of the educational process and outcomes of higher education institutions has become a major goal of higher education authority worldwide [18].

The main stakeholders of medical schools are the patients who seek care at their teaching hospitals and the wider community they serve. When medical schools are transparent in how they communicate their curriculum, teaching methods, and student assessments, it helps build trust with these key stakeholders. Publishing clear curriculum statements outlining teaching and assessment methods is critical for ongoing monitoring, review and evaluation [19]. Such data will facilitate the transfer of students between different medical schools within a country or internationally. Open communication and data sharing with the public and other stakeholders through transparency is an essential part of accountability and quality improvement in medical education that ultimately benefits the communities these institutions serve.

The transparency initiative should make available all details regarding accreditation procedures and requirements.This should be disclosed more abundantly and more easily accessed to all stakeholders and thus committing everyone to the concept of shared responsibility [8]. In this way medical schools may push towards continuous quality improvement (CQI) and engage with the public to promote a better healthcare service.

If transparency is missing, trust also follows. Medical schools must aspire to be sufficiently trusted among their students, faculty, staff, other schools, and most importantly, their community. Transparency increases community awareness of the quality of educational programs for medical schools.

Recent developments in assessment and curriculum development have emphasized the importance of involving the population in health professions education [20]. If we want civil society to peer-review medical school and engage in the training of medical students as well as in curriculum development, then transparent information must be promoted by medical institutions to increase public accountability [18].

NAQAAE’s mission statement asserts that to ensure quality in any educational institution, its program must be consistent with the institution’s recognized mission statements and stated goals. An institution’s mission and curriculum help build trust in its graduates within the community. In addition, accreditation criteria require that institutions have internal quality assurance systems that provide annual reports on institutional development and improvement. This demonstrates the importance of transparency and information disclosure for building stakeholder trust and ensuring accountability. By making reports on its quality assurance processes and outcomes public, an institution signals that it meets high standards and continuously strives for improvement [6].

The concept of openness to governance must be accepted in the culture of Egyptian medical schools with the aim of pushing these schools towards working on improvements and discovering their weaknesses and risks of curricula in a highly collaborative environment. Such a change in mindset will enhance learning within institutions as well as focus on developing healthcare services and enriching human health.

Egyptian medical schools frequently celebrate their improved university rankings globally. The World Universities Ranking is mainly based on internationally published research scholarships. This process is unfair to assess the success of a medical school in relation to an undergraduate medical education. On the other hand, although these ratings can skew the message of medical schools, they can still affect the public.

Medical school websites should provide details about their teaching hospitals, patient volumes, types of clinical rotations available and student/patient ratios to demonstrate they meet international standards for hands-on clinical training. A lack of transparency regarding the teaching hospital and clinical training resources could indicate potential shortcomings and limitations in a medical school’s ability to provide a full range of clinical experiences for its students [11].

While there are many benefits to transparency in medical schools, some leadership may be hesitant due to potential challenges. Concerns include public interference in decision-making that could negatively impact student recruitment, staff morale and funding. Leaders may worry that publicizing weaknesses could have financial implications that hamper hiring and improvements. Competition between medical schools could intensify instead of become more collaborative [18]. However, the need to shape public opinion can motivate schools to be more transparent in order to gain trust and recognition of their important work. Total secrecy is unlikely to build long-term stakeholder trust and confidence. Medical schools can prioritize transparency related to educational outcomes, policies, operations and broader institutional performance while restricting disclosure of sensitive data that could infringe on privacy, confidentiality or potentially harm the institution. With careful planning and the right balance, transparency and privacy can coexist within an accountable and trustworthy institution. Proper risk management practices can help ensure the institution only discloses appropriate information in constructive ways that support - rather than undermine - its core mission [21].

## Conclusion and recommendations

While transparency offers many benefits for medical schools and the communities they serve, institutions must also consider the risks and balance transparency with the need for privacy and confidentiality. With careful planning, proper policies and effective risk management, medical schools can implement transparency in constructive ways that harness its full potential while mitigating threats. Total secrecy is unlikely to build long-term trust, but excessive disclosure without strategy could also be problematic. The optimal model of transparency for medical schools likely involves engaging the public as partners, sharing appropriate information that demonstrates institutional accountability and quality improvement, while simultaneously protecting sensitive data. Transparency must be implemented with a focus on its ultimate purpose: to enable better outcomes for students, patients and society through more open and collaborative relationships with key stakeholders [22].

Transparency is a means to an end, not an end in itself. When implemented constructively and managed responsibly, transparency can enable medical schools to better fulfill their vital role in society through stronger relationships with stakeholders built on openness, trust and mutual understanding.

## Limitations

This study provided a relative index of the data but was only able to look at the extent of transparency of Egyptian medical schools’ websites. However, no comparison has been made globally to check other international school websites. Additional studies may be important to establish the level of transparency of Egyptian medical schools compared to similar international medical schools in this respect. Further general education may be necessary to ensure that accreditation requirements are provided in detail and that institutions comply with them.

## Data Availability

The data supporting the findings of this study are available from the websites of the medical schools; but limitations apply to the availability of the analyzed data, which were used under license for the current study, and so are not available to the public. However, data are available from the corresponding author on reasonable request via email.

## Abbreviations

ESME: Egyptian Society for Medical Education
NAQAAE: National Authority for Quality Assurance and Accreditation of Education
REDCap: Research Electronic Data Capture
WFME: World Federation for Medical Education
KPIs: Key Performance Indicators
